# The effect of sex and estrus cycle stage on optogenetic spreading depression induced migraine-like pain phenotypes

**DOI:** 10.1186/s10194-023-01621-1

**Published:** 2023-07-19

**Authors:** Andrea M. Harriott, Angel Waruinge, Viola Appiah-Danquah, Leah Berhanu, Andreia Morais, Cenk Ayata

**Affiliations:** 1 Neurovascular Research Laboratory, Department of Radiology, Massachusetts General Hospital, Boston, MA USA; 2 Department of Neurology, Neurovascular Research Laboratory, Massachusetts General Hospital, Boston, MA USA; 3 Emmanuel College, Boston, MA USA; 4 Cambridge Rindge and Latin School, Boston, MA USA

**Keywords:** Optogenetic spreading depolarization, Periorbital allodynia, Sex and hormone differences

## Abstract

**Background:**

Migraine is more prevalent in females, raising the possibility that sex and gonadal hormones modulate migraine. We recently demonstrated that minimally invasive optogenetic spreading depolarization (opto-SD) elicits robust periorbital allodynia. The objective of this study was to test the hypothesis that opto-SD induced migraine-like pain behavior is worse in females and varies during the estrus cycle.

**Methods:**

Single or repeated opto-SDs were induced in male and female adult Thy1-ChR2-YFP transgenic mice. Von Frey monofilaments were used to test periorbital mechanical allodynia. Mouse grimace was also examined under increasing light intensity to quantify spontaneous discomfort and light-aversive behavior. Vaginal smears were obtained for estrus cycle staging at the end of behavioral testing.

**Results:**

A multi-variable regression analysis was performed using a male and female cohort to test the effect of independent variables on periorbital allodynia. Opto-SD predicted lower periorbital thresholds as compared with sham stimulation (*p* < 0.0001). Additionally, female sex predicted lower periorbital thresholds compared with males (*p* = 0.011). There were significant interactions between opto-SD and time (interaction *p* = 0.030) as animals tended to recover from opto-SD allodynia over time, and between sex and time (*p* = 0.020) as females tended to take longer to recover. Proestrus, estrus (PE) and metestrus, diestrus (MD) stages were combined to represent high versus low circulating estradiol relative to progesterone, respectively. Multi-variable regression revealed an effect of estrus cycle (*p* = 0.015) on periorbital thresholds. In the sham group, PE had lower thresholds than MD. However, there was no interaction between opto-SD and the estrus cycle (*p* = 0.364). Grimace scores were also examined at incremental light intensities. There was an effect of opto-SD (*p* < 0.0001), light intensity (*p* = 0.001) and estrus cycle (*p* = 0.024) on grimace without interaction among them (three-way ANOVA).

**Conclusions:**

Female sex and estrus stages with high circulating estradiol relative to progesterone lower trigeminal pain thresholds and augment photosensitivity. In females, opto-SD increased pain behavior and photosensitivity irrespective of the estrus stage.

**Graphical Abstract:**

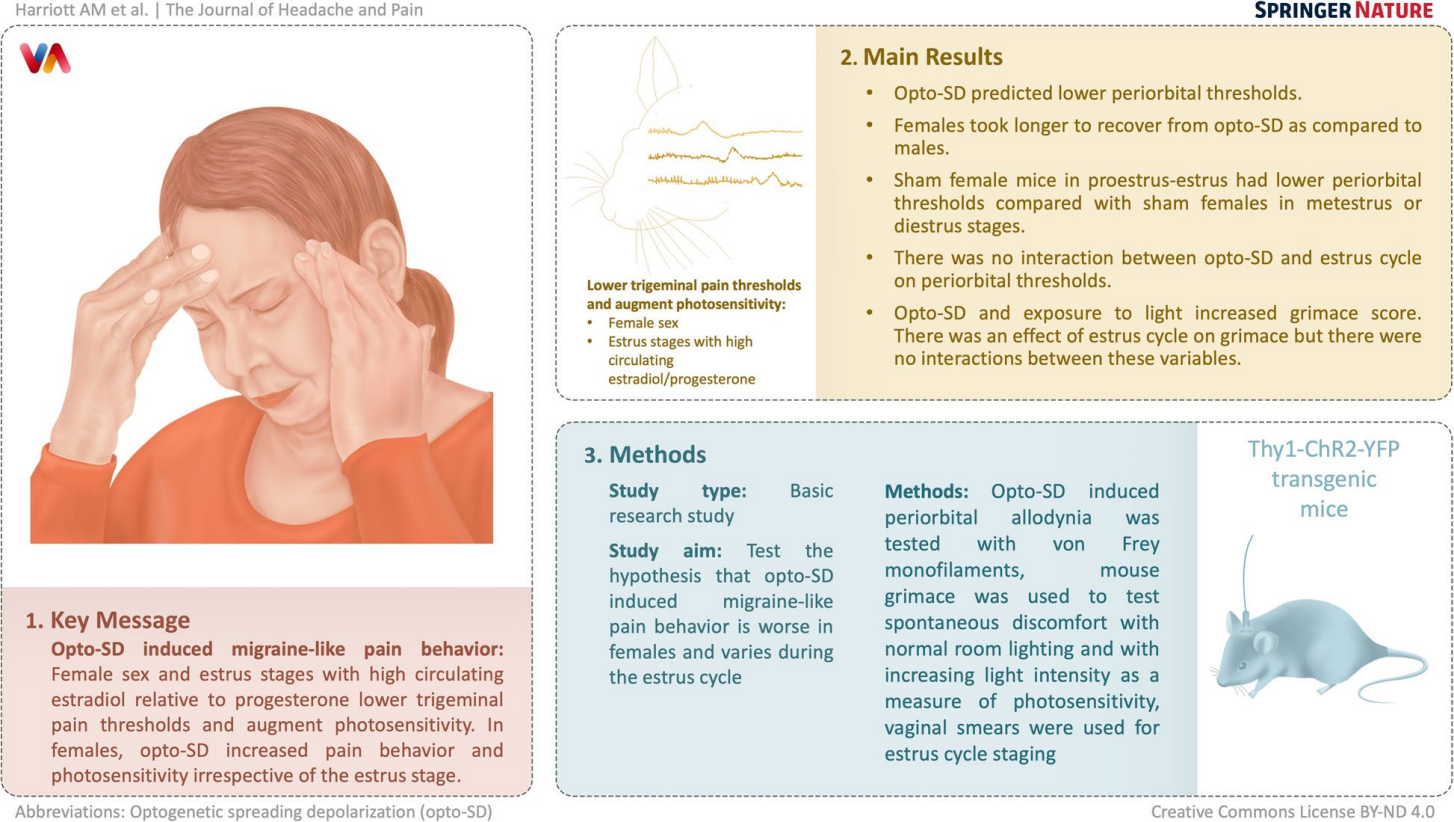

## Background

Migraine affects 20–30% of women between the ages of 20 and 40 and is three times more common in women as in men [28]. Migraine tends to start after menarche, vary in intensity with menstruation, and abate after menopause, implicating gonadal hormones in the migraine-sex disparity and raises the possibility that gonadal hormones may play an important role in disease phenotype [23]. There may also be a role for chromosome and/or hormone-chromosome interactions in the migraine-sex disparity.

Recent studies have uncovered sex differences in rodent models of migraine without aura (MwoA) [1–4, 36, 40, 43, 47]. Dural calcitonin gene-related peptide (CGRP) application produced facial allodynia and spontaneous grimace in adult female but not male rats and mice [3]. Similarly, dural application of inflammatory mediators (histamine, serotonin, bradykinin, prostaglandin) produced greater and longer lasting periorbital allodynia in females compared with males [43]. In a model of repeated intraperitoneal administration of nitroglycerin (NTG) 10 mg/kg, females had longer-lasting NTG-induced allodynia than males [1, 36]. These studies suggest females have more severe pain outcomes in rodent models of MwoA.

Migraine with aura (MwA) is a distinct subtype of migraine with characteristic visual disturbances accompanying headache. MwA is associated with higher rates of cutaneous allodynia compared with MwoA [8, 27, 29–31], comorbidities, and disability [29]. While MwA is more prevalent in females, there has been a paucity of preclinical studies examining the impact of sex in preclinical models of MwA and fewer studies examining the influence of fluctuations in normal cycling female gonadal hormones on the magnitude of pain behavior. Unlike MwoA, MwA is less associated with estradiol withdrawal during the start of menses and less likely to improve or remit during pregnancy [22]. Moreover, new onset migraine during pregnancy is more often MwA [12, 15, 22]. Lastly, the use of oral contraceptives is more likely to worsen MwA than MwoA attacks [22]. These data suggest divergent effects of exogenous and endogenous gonadal hormones, most probably estradiol, on the ability to trigger and exacerbate the severity of headaches associated with MwA [22, 32] and justify examining sex and hormone mechanisms separately in the MwA subtype.

Cortical spreading depolarization (SD), a slow wave of neuronal and glial excitation, is the likely cause of MwA and used as a preclinical model. We recently employed a minimally invasive optogenetic approach to induce SD (opto-SD), which produced robust trigeminal mechanical allodynia, grimace behavior and enhanced anxiety-related behavior, without the confounding effects of craniotomy or durotomy [24]. The objective of this study was to test the hypothesis that minimally invasive opto-SD-induced migraine-relevant behavior is modulated by female sex and estrus cycle stage. Nulliparous adult mice typically have a 4–5 day estrus cycle during which 17βestradiol (E2) levels rise between proestrus and estrus stages, peaking during ovulation. Between proestrus and estrus stages, progesterone (P4) levels are low. The E2:P4 ratio is therefore higher during proestrus-estrus. The converse occurs during metestrus and diestrus stages. During transition from metestrus to diestrus, there is a diminution in E2 levels while P4 levels continue to rise producing a low circulating E2:P4 concentration. Therefore, proestrus plus estrus (PE) and metestrus plus diestrus (MD) cycle stages represent high versus low circulating E2 levels relative to progesterone, respectively [20]. These natural variations, while not exactly recapitulating the human menstrual cycle, can be used to examine how gonadal hormones may influence pain states in a sex-specific manner.

## Methods

Mice were housed under 12-12 h light/dark cycles in temperature-controlled rooms. Food and water were given freely. Experiments were performed at the Massachusetts General Hospital Neurovascular Research Unit.

### SD induction

Transgenic mice (*n* = 61 male, 121 female; 4–8 months old, 25-35 g) expressing channelrhodopsin-2-YFP fusion protein under the thymus cell antigen-1 promoter (ChR2^+^; B6.Cg-Tg(Thy1-COP4/EYFP)9Gfng/J – Line 9) were randomly assigned to opto-SD or sham groups. Under brief isoflurane anesthesia with 70% N_2_O/30% O_2_, the head was fixed on a stereotaxic frame, the scalp incised, and a glass coverslip fixed to the skull for transparency using cyanoacrylate and C&B Metabond 1 week prior to behavior testing. A 400 µm fiberoptic light source was positioned over the motor cortex for opto-SD induction using a square pulse of 470 nM light for 10 s at 10mW (Thorlabs, Newton, NJ, USA). Optogenetic stimulation was used to induce either a single SD once or repeatedly every other day for a total of 7 SDs to examine the effect of repeated SD on behavior. SDs were confirmed using characteristic reflectance changes propagating across the cortex on intrinsic optical signal difference images using a webcam (OT-HD, Opti-TekScope, Chandler, AZ, USA) interfaced with MATLAB as previously described [14]. Animals in the sham control group underwent coverslip placement and brief exposure to anesthesia and head fixation without opto-SD stimulation. We recently showed that single and repeated opto-SD produced trigeminal pain behavior in mice [24]. These previously collected data from 61 male and 47 female mice were used to examine the effect of sex and the interaction between sex and time after SD on periorbital mechanical thresholds. Data from an additional 74 females were used to examine the effect of estrus cycle stage.

### Estrus cycle staging

To determine the estrus stage, vaginal smears were examined within 30 min of behavior testing using lavage. Approximately 25–40 µl ddH2O was expelled and aspirated from a pipette tip at the opening of the vaginal canal. The final aspirate was placed on a coverglass, smeared, and stained with crystal violet for inspection with light microscopy (Fig. 5). Proestrus was identified as smears with predominantly large, nucleated cells and estrus as cornified epithelial cells. Diestrus and metestrus were distinguished by the presence of leukocytes with smears from animals in diestrus also containing nucleated epithelial cells [34].

In addition to vaginal smears, we tested whether infrared temperature (IRT) imaging of surface body temperature using an uncooled microbolometer vanadium oxide infrared long-wavelength (8–14μm) sensor can reliably delineate the estrus cycle (*n* = 4). The infrared thermal imaging camera (220 × 160, temperature detection range of -20ºC to + 300ºC; IR HTI Thermal Imager, Xintai Instrument Co., Ltd.) accurately measured water temperatures against a mercury thermometer. The microbolometer absorbs incident infrared thermal radiation emitted from the mouse. The surface region used was the periorbital/head region. Surface temperature may differ depending on surface region used. There are diurnal fluctuations in core body temperature [26] which may contribute to some variability but that is unlikely to explain differences between estrus cycle stage since the surface temperature readings were obtained within the same 3-h time period in the mid-afternoon. Three to five values per animal per cycle stage were averaged and taken with a field of view that included the head at approximately 15 cm distance. Vaginal smears were collected after temperature values were recorded. This was repeated every other day for 10 days in 2 animals and 12 days in the other 2 animals for 22 averaged readings and vaginal smears.

### Behavior assessment

Mice were acclimated to behavioral rooms and test chambers before behavior assessments. Behavior testing was performed between late afternoon-early evening on each test day. Periorbital allodynia was assessed 1 h after a single opto-SD or 2 days after repeated opto-SD using mechanical thresholds with sequential ascending calibrated von Frey monofilaments (0.008 to 0.4 g, Stoelting Co, Wood Dale, IL, USA), by manual application to the superior medial right and left periorbital region as previously reported [16, 19] and values for the right and left side were averaged [24]. An avoidance response to mechanical stimulation was determined by observing the animal stroking the ipsilateral face with the forepaw or brisk head turn away from the stimulus.

Mouse grimace scale (MGS) was used to assess spontaneous discomfort in mice. To assess photosensitivity, MGS was examined in room light (75 lx) and with increased light exposure between 400 – 8400 lx. Following periorbital mechanical testing, a cohort of 45 animals remained in the restrainer device. We used a white LED light source with 6 dial settings to deliver light intensities between 400 – 8400 lx. Animals were positioned 10 cm from the light source. There was no change in ambient heat from the light source and light was measured with a photometer (URCERI MT-912 Digital Illuminance Light meter). Animals were exposed to each light intensity starting with room light for 60 s. Face expressions were scored from not present = 0, moderate = 1, and severe = 2, for each of 4 action units (orbital tightening, nose bulge, cheek bulge, and whisker position) as previously described [24]. Frames were captured from video at 15, 30 and 45 s for each light intensity and grimace scores from each frame were averaged. Grimace scores were then averaged for each light intensity. Averaged values for animals in SD and sham groups are presented across light intensities.

### Statistical and data analysis, and experimental rigor

For all groups, animals were randomly assigned to opto-SD stimulation or sham control groups. The estrus cycle stage was unknown to the examiner during the behavior assessment. All experiments were blinded to SD or sham group allocations or estrus cycle stage until after data collection. Identifying cage cards were replaced with random letter-number cards prior to behavior testing. There were no other markings or identifiers, and animals were unblinded only after data collection. Animals were randomly picked from cages in alternating fashion for SD or sham groups. Vaginal smears were collected after behavior testing. Therefore, the investigator was blinded to the estrus stage at the time of behavior testing. We selected periorbital allodynia as the primary endpoint and powered the study accordingly. Based on prior experience, sample sizes aimed to detect a 20% reduction in allodynia per estrus cycle stage group (α = 0.05, power = 80%, *n* = 12/group). Statistical analyses were conducted using GraphPad Prism 7.0 and indicated in the results section and figure legends for each dataset. Two-tailed *p *< 0.05 was considered statistically significant. Data were presented as mean ± standard error (SEM). For heteroscedastic data, values were rank-transformed prior to parametric data analysis.

## Results

### Sex differences in opto-SD-induced periorbital allodynia

**Fig. 1:**
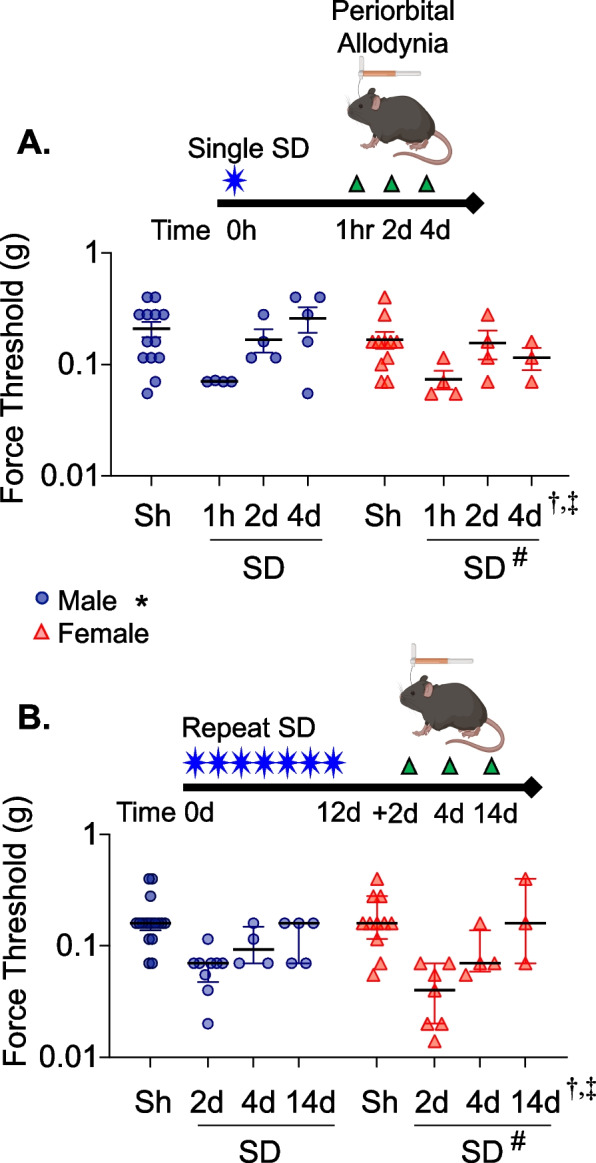
Single and repeated opto-SD on periorbital mechanical allodynia in male and female mice. Periorbital thresholds were tested at 1 h, 2 days and 4 days after single SD or sham exposure (**A**), or 2, 4 and 14 days after the last repeated SD (**B**). Individual force threshold data (grams) from single SD groups for males (blue) and females (red) are shown. Opto-SD lowered periorbital mechanical thresholds (^#^*p* < 0.0001 vs. sham). Female sex was also associated with lowered periorbital thresholds (*p = 0.011 vs. male). There were significant interactions between opto-SD and time of behavior test (^†^*p* = 0.030) as animals recovered from opto-SD induced allodynia over time and between sex and time of behavior test (^‡^*p* = 0.020) as females seemed to take longer to recover from opto-SD

Periorbital mechanical thresholds were measured in 61 male and 47 female adult mice following a single opto-SD or 7 repeated opto-SDs once every other day and compared to sham controls (Fig. 1). Thresholds were determined 1 h, 2 or 4 days after a single opto-SD, or 2, 4 or 14 days after the last of 7 opto-SDs, in separate cohorts. A multi-variable regression analysis was performed on the pooled cohort to test the effect of independent variables including sex, sham versus opto-SD, time of behavior testing, and single or repeated SD. We also included interactions between opto-SD and time of behavior testing, and sex and time of behavior testing on periorbital mechanical thresholds in the model. As previously demonstrated [24], opto-SD predicted lower periorbital mechanical thresholds (*p* < 0.0001), which recovered over time (interaction between opto-SD and time after SD, *p* = 0.030). There was a significant effect of sex on periorbital allodynia (*p* = 0.011). Females had lower periorbital mechanical thresholds compared with males (Table 1). We also found a significant interaction between sex and the timing of behavior testing on peripheral allodynia (*p* = 0.020). Females took longer to recover from opto-SD allodynia than males (Fig. 1). The effect of female sex on recovery from opto-SD is more notable in the single opto-SD group. Following single opto SD, testing at 4 days in males produced periorbital mechanical threshold that was closer to sham (male single opto-SD at 4 days: 0.259 ± 0.068 g) while in females the threshold at this time point still appeared low (female single opto-SD at 4 days: 0.115 ± 0.026 g).

**Table 1:** Effect of sex on opto-SD induced periorbital thresholds using multivariable linear regression

Independent variables	Beta	95% CI		STE	|t|	*p*
**Sex**	-18.50	-32.71	-4.29	7.16	2.58	0.011
**Opto-SD**	-36.53	-50.48	-22.58	7.03	5.20	< 0.0001
**Time of behavior test**	-0.67	-2.58	1.22	0.96	0.71	0.481
**Single or Repeated stimulation**	-15.01	-25.82	-4.19	5.45	2.75	0.007
**Opto-SD x Time of behavior test**	2.60	0.26	4.93	1.18	2.20	0.030
**Sex x Time of behavior test**	3.10	0.49	5.71	1.31	2.36	0.020

Periorbital allodynia = β_0_ + β_1_(Sex) + β_2_(Opto-SD) + β_3_(Time of behavior test) + β_4_(Single or Repeated stimulation) + β_5_(Opto-SD x Time of behavior test) + β_6_(Sex x Time of behavior test)

### Impact of estrus cycle on pain behavior

Mice were found to be in proestrus (*n* = 8 sham, *n* = 7 SD), estrus (*n* = 9 sham, *n* = 12 SD), metestrus (*n* = 8 sham, *n* = 5 SD), or diestrus (*n* = 4 sham, *n* = 7 SD) using vaginal lavage immediately after behavior testing [34]. For data analysis across estrus cycle stages, proestrus and estrus stages were combined, and diestrus and metestrus stages were combined to represent high versus low estradiol to progesterone levels and to increase statistical power [34].

### Estrus cycle stage on periorbital allodynia

**Fig. 2:**
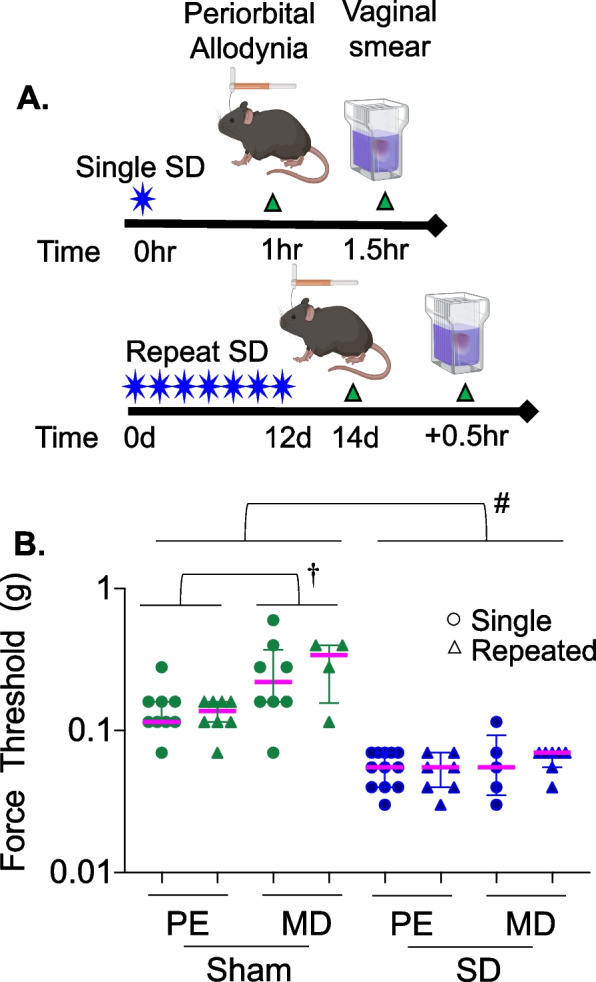
Estrus cycle and opto-SD-induced periorbital allodynia. Periorbital allodynia was tested either 1 h after single opto-SD (circles) or 2 days after the last day of repeated opto-SD (triangles). Vaginal smears were collected after behavior testing, (**A**). In multiple linear regression analysis, there was a significant effect of opto-SD (^#^*p* < 0.0001 vs sham) and estrus cycle stage (*p* = 0.015) on periorbital thresholds, but no interaction between the two. Using two-way ANOVA of rank transformed data from SD and sham groups, and Sidak test for multiple comparisons, metestrus-diestrus (MD) sham females had higher periorbital thresholds (^†^*p* = 0.029 vs PE sham females). There was no effect of estrus stage on periorbital thresholds following opto-SD as both MD and PE animals had significant reductions in mechanical thresholds following opto-SD, (**B**)

The effect of estrus cycle stage on periorbital mechanical threshold was tested at 1 h after a single opto-SD or 2 days after repeated opto-SDs when allodynia was most robust in each model. Multi-variable linear regression of rank-transformed data was used to test the effect of estrus cycle stage and opto-SD on periorbital allodynia. Opto-SD lowered periorbital mechanical thresholds compared with sham stimulation (*p* < 0.0001). The MD stage showed higher periorbital thresholds than PE (*p* = 0.015). Allodynia after a single versus repeated opto-SDs did not differ at the selected time points of assessment (*p* = 0.865, effect estimate 0.42, 95% CI -4.5 to 5.4). Sham animals in MD had higher periorbital thresholds compared with those in PE [MD sham (*n* = 12): 0.275 ± 0.044 g; PE sham (*n* = 17): 0.138 ± 0.012 g, *p* = 0.029, two-way ANOVA on ranked data with Sidak test]. However, irrespective of the estrus cycle stage, opto-SD produced a robust drop in periorbital mechanical thresholds with no interaction between opto-SD and estrus cycle stage (*p* = 0.364, Table 2). Opto-SD triggered periorbital allodynia compared to their respective sham cohorts regardless of the cycle stage [MD opto-SD (*n* = 19): 0.062 ± 0.007 g; PE opto-SD (*n* = 12): 0.055 ± 0.003 g, Fig. 2].

**Table 2:** Effect of estrus cycle stage on opto-SD induced periorbital mechanical thresholds

Independent variables	Beta	95% CI		STE	|t|	p
**Opto-SD**	-26.24	-32.51	-19.96	3.13	8.38	< 0.0001
**Estrus cycle (PE or MD)**	8.94	1.85	16.03	3.54	2.53	0.015
**Single or Repeated stimulation**	0.42	-4.54	5.39	2.47	0.17	0.865
**Estrus cycle x Opto-SD**	-4.58	-14.61	5.45	5.00	0.91	0.364

Periorbital allodynia = β_0_ + β_1_(opto-SD) + β_2_(Estrus cycle) + β_3_(Single or Repeated stimulation) + β_4_(Estrus cycle x opto-SD)

### Estrus cycle stage on mouse grimace and photosensitivity

In addition to periorbital allodynia, we examined opto-SD-induced spontaneous discomfort using mouse grimace score in female mice (*n *= 21 sham, *n* = 24 SD, three-way ANOVA: opto-SD vs sham, estrus cycle, light intensity). Opto-SD significantly increased grimace in female mice, suggesting SD-induced spontaneous discomfort (*p* < 0.0001). There was a significant effect of estrus cycle on grimace scores (*p* = 0.024). There was no interaction between opto-SD and estrus cycle stage (*p* = 0.174).

**Fig. 3:**
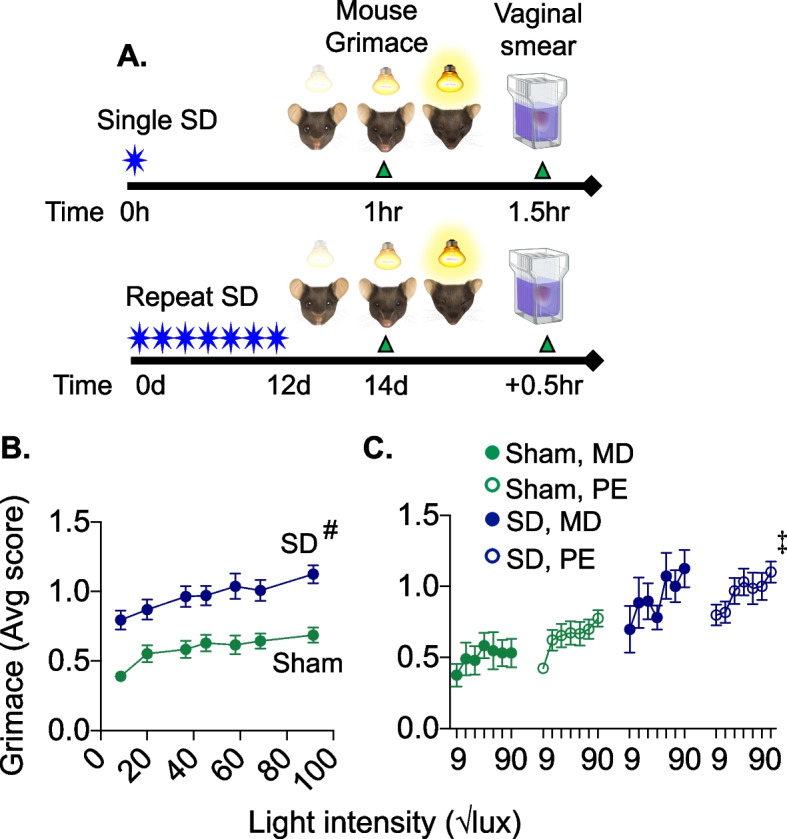
Estrus cycle and opto-SD-induced grimace. Mouse grimace score (MGS) was determined at 75 lx (room lighting) and with increasing light intensity between 400–8400 lx, (**A**). Data shown in (**B**) and (**C**) are grimace scores plotted against increasing light intensity using the square root of lux values. Fit with a simple linear regression, there is no difference in slopes between sham and opto-SD grimace-light intensity curves (*F* = 0.30, *p* = 0.585). Opto-SD produced a significant elevation in the grimace-light intensity curve (*F* = 117, ^#^*p* < 0.0001 vs sham, B). Using three-way ANOVA, there was a significant effect of opto-SD (*F* = 90.82, *p* < 0.0001), light intensity (*F* = 3.82, *p* = 0.001) and estrus cycle stage (*F* = 5.19, *p* = 0.024) on grimace score (C). Grimace-light intensity curves demonstrated significantly non-zero slopes^‡^ for animals in PE sham (*F* = 11.17, *p* = 0.001), MD opto-SD (*F* = 5.56, *p* = 0.022), and PE opto-SD (*F* = 8.04, *p* = 0.005)] groups. However, MD sham animals did not have a significant slope of the grimace-light intensity curve, suggesting this group did not experience as much photosensitivity (*F* = 1.27, *p* = 0.266). There was no significant difference in the elevation (*F* = 0.23, *p* = 0.635) or slope (*F* = 0.35, *p* = 0.553) within the opto-SD groups between MD versus PE cycle stages

Exposure to increasing light intensity above room light (between 400 and 8400 lx) increased the grimace score in both sham and opto-SD stimulated animals (*p* = 0.001). When grimace-light intensity curves were fit with a simple linear regression, there was a similar slope in the SD and sham groups (*F* = 0.30, *p* = 0.585; Fig. 3). Opto-SD produced an upward shift in the grimace light intensity curve compared with sham stimulation (*F* = 117, *p* < 0.0001, Fig. 3). There was no interaction between light intensity and estrus cycle stage (*p* = 0.949). However, when grimace-light intensity curves were separated by estrus cycle and fit with simple linear regression, PE sham (*F* = 11.17, *p* = 0.001), MD opto-SD (*F* = 5.56, *p* = 0.022), and PE opto-SD (*F* = 8.04, *p* = 0.005) groups demonstrated significant non-zero slopes. The grimace-light intensity curve in sham animals in MD did not have a significant non-zero slope (*F* = 1.27, *p* = 0.266). Both MD and PE opto-SD groups demonstrated upward shifts in the grimace-light intensity curves compared to sham animals (Fig. 3).

### Repeated SD does not impact normal cycling

**Fig. 4:**
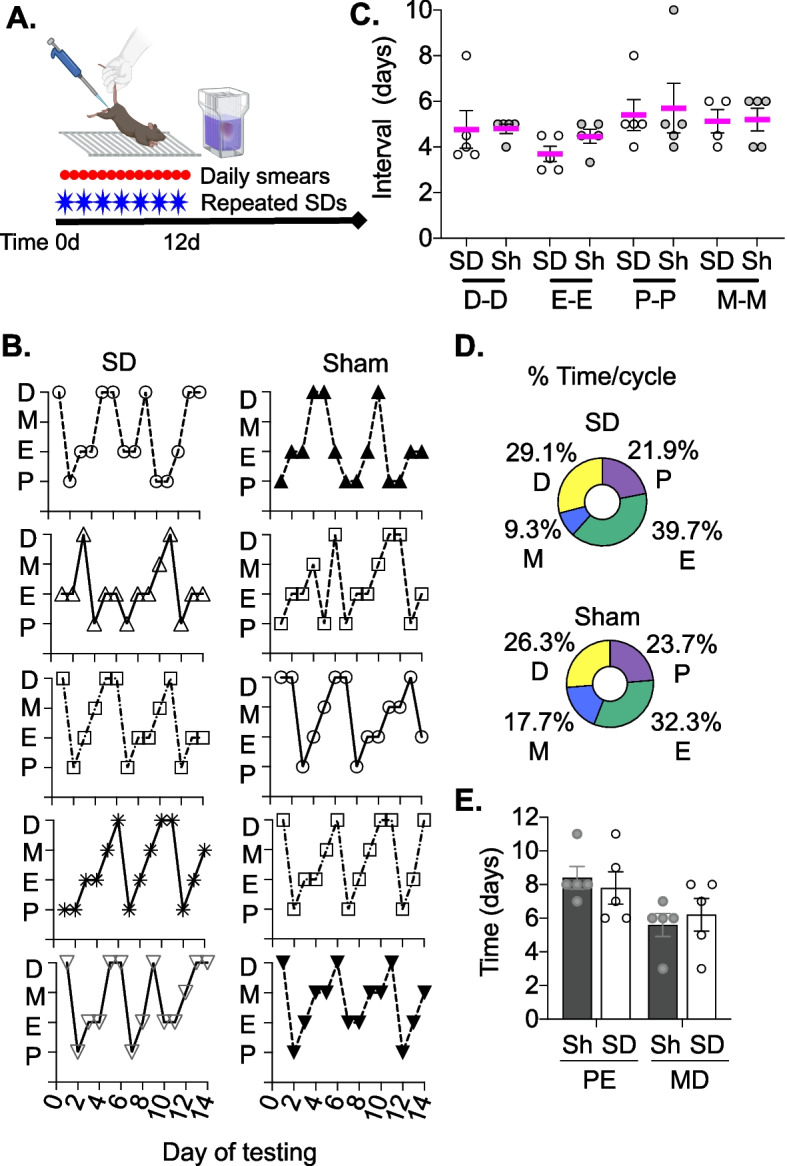
Repeated SD does not affect estrus cycling. Vaginal smears were collected daily for 2 weeks while mice underwent repeated opto-SD × 7 SDs (*n* = 5) and compared to sham mice (*n* = 5, **A**). Cycles from individual mice are shown in (**B**) for opto-SD and sham animals. The time in days between consecutive diestrus, estrus metestrus or proestrus stages were averaged in sham and opto-SD groups to estimate the duration of the estrus cycle. There was no effect of repeat opto-SD on duration of the cycle as measured by interval days between consecutive cycle stages in opto-SD versus sham animals (cycle stage used: *F* = 2.02, *p* = 0.131; SD: *F* = 0.44, *p* = 0.514, **C**). More time was spent in estrus followed by proestrus, diestrus and metestrus cycle stages for both groups, (**D**). When data were grouped in PE and MD stages, there was no difference in the total number of days spent in PE or MD stages between sham and opto-SD groups over the 2 weeks tested (*F* = 0, *p* > 0.9999, two-way ANOVA, E)

To determine if repeated opto-SD disrupted cycling, daily vaginal smears were collected in a separate cohort of mice during 7 repeated opto-SDs induced every other day and compared to sham animals (*n* = 5 each sham and opto-SD; Fig. 4). The time interval between cycle stages was determined for diestrus, estrus, proestrus and metestrus. There was no effect of estrus cycle stage (*p* = 0.131) or SD stimulation (*p* = 0.514, two-way ANOVA) on the interval time between cycle day which ranged from 3.70 ± 0.34 days (estrus) to 5.40 ± 0.68 days (proestrus) in the SD group and 4.47 ± 0.30 days (estrus) to 5.70 ± 1.1 days (proestrus) in the sham group. Sham and opto-SD animals spent more time in estrus stage compared to other phases of the cycle. Animals were captured in diestrus and metestrus less frequently. There was no significant difference in the time in days spent in PE or MD between sham versus opto-SD groups (*p* > 0.9999 two-way ANOVA).

### Assessment of the estrus cycle stage

**Fig. 5:**
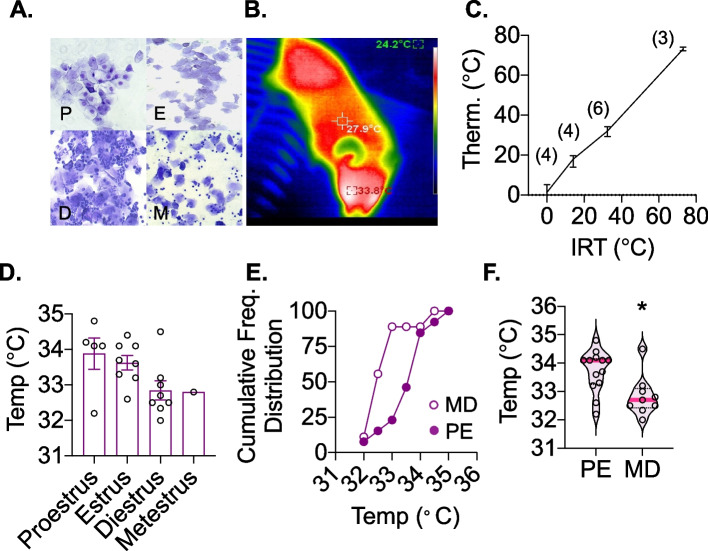
Estrus cycle stage and surface infrared thermography (IRT). Crystal violet stain of vaginal smears for proestrus, estrus, diestrus, and metestrus cycle stages (labeled P, E, D, M respectively in the figure) are shown in (**A**). IRT image of a mouse using a vanadium oxide microbolometer infrared sensor thermal imaging camera is shown in (**B**). Thermal images displayed three temperatures: the highest temperature which imaged the periorbital/head region (red: 33.8°C), the lowest temperature which was of the cage lid (green), and the temperature at the central point of the image (white). Surface temperature of water from a mercury thermometer corresponds with temperature detected using IRT across a wide range of temperatures, (**C**). IRT temperature for *n* = 4 animals taken across the estrus cycle stage demonstrates the lowest temperature during diestrus and metestrus, (**D**). The cumulative distribution of temperatures for animals in proestrus-estrus (PE, filled circles) and metestrus-diestrus (open circles) shows a right shift in temperatures for animals in PE cycle stage, (**E**). Median temperature during PE (34.1°C, *n *= 13) was significantly higher than during MD (32.7°C, *n* = 9, *p = 0.017, Mann Whitney U), (**F**)

Given the lower rate of capture for animals in MD, we investigated if noninvasive surface infrared thermography (IRT) could be used in future studies to assist in predicting the cycle stage. Thermal imaging was performed using an uncooled microbolometer vanadium oxide infrared long-wavelength (8–14 μm) sensor. Thermal images displayed three temperatures: the highest temperature which was around the periorbital/head region, the lowest temperature which was of the cage lid, and the temperature at the central point of the image (Fig. 5). A cohort of 4 mice were monitored with concurrent thermal imaging and estrus cycle staging using repeated vaginal smears over 10–12 days. Animals in the proestrus stage had the highest surface IRT detected temperature (33.88 ± 0.4ºC) compared with animals in diestrus (32.84 ± 0.8ºC). When the cumulative distribution of IRT temperature values from PE cycle stages were plotted with those from MD cycle stages, there was a right shift in the curve of surface temperatures during PE and significantly higher average IRT surface temperature during PE compared with MD stage (*p* = 0.017, Mann Whitney U, Fig. 5).

## Discussion

In this study, we show that female mice have more pronounced opto-SD-induced periorbital allodynia than males after accounting for the time of behavior testing after opto-SD. We also show that sham-control females in the PE stage (i.e., higher circulating E2:P4) display lower periorbital thresholds and higher photosensitivity than those in the MD stage. These data suggest that normal cycling female mice have hormone-dependent variations in pain behavior, even without SD stimulation. Opto-SD produced a robust periorbital allodynia and amplified the grimace-light intensity response irrespective of the estrus cycle stage. Therefore, in the presence of SD, estrus stage may have less impact on the magnitude of pain behavior outcome. An alternative explanation is that opto-SD is not sensitive to detect the impact of normal cycling hormone effects on pain behavior. To our knowledge, this is the first study of the impact of estrus cycle stage on opto-SD induced migraine-relevant behaviors.

That we detected sex differences in periorbital allodynia when considering time of behavior testing after opto-SD is consistent with results from other studies [1, 36, 43]. In our prior study [24], sex differences were not detected when time after opto-SD was not taken into account [21]. In both CGRP and inflammatory mediator migraine models as well as in the NTG model, females displayed greater allodynia and longer recovery time from allodynia as compared to males [1, 43]. These data demonstrate that, while preclinical models do not completely recapitulate the complexity of the human condition; there is evidence for female susceptibility to worse pain phenotypes using migraine preclinical models.

Compared to MD, females in PE appeared to have relatively lower periorbital mechanical thresholds and higher photosensitivity. These data suggest that baseline pain thresholds are dependent on fluctuations in gonadal hormones. Modulation of pain in migraine relevant neural circuits by circulating estrogens may explain this enhanced baseline pain behavior. Estrogen receptors (ERα and β) are located both peripherally and centrally in the trigeminal system and likely play important roles in nociception. For example, ERα is expressed in neuropeptide containing trigeminal nociceptors. During estrus, there is an increase in expression of ERα. There is also an increase in expression of galanin and neuropeptide Y during estrus and proestrus as compared to diestrus [37]. Both ERα and β are in small diameter Na_v_1.7 containing trigeminal neurons. In one study using female Sprague Dawley rats, cranial mechanical thresholds were lower in proestrus when estradiol levels were higher compared to other phases of the estrus cycle. Complete Freund’s adjuvant (CFA) injected into the bilateral temporomandibular joint (TMJ) as a model of TMJ disorder produced facial mechanical allodynia which was exacerbated in ovariectomized rats that received estradiol replacement compared with vehicle replacement. Furthermore, inhibition of both ERα and β in normal cycling females with systemic administration of ICI 182,780 partially prevented CFA-induced TMJ mechanical hypersensitivity. These data suggest that estrogens may be important not only for changes in baseline thresholds across the estrus cycle, but also for behavioral responses in particular trigeminal pain models [6].

That we did not find greater opto-SD-induced periorbital allodynia or grimace during PE versus MD or an interaction between estrus stage and SD in our analyses suggest that SD-induced migraine-related pain phenotypes do not depend on fluctuations in gonadal hormones during the estrus cycle, the impact of gonadal hormones may be stronger in non-SD migraine models, the SD model may not be sensitive to resolve differences in pain behavior mediated by fluctuations in hormones during the estrus cycle, and/or gonadal hormones may act on upstream events like SD susceptibility rather than downstream events occurring after SD induction. To support the last point, there is data demonstrating an effect of sex and gonadal hormones on SD susceptibility. In one study, the volume of 1 M KCl needed to evoke SD was lower in female as compared to male mice. The electrical stimulus needed to evoke SD in female mice was likewise lower than in males, with no sex difference in SD properties (amplitude, velocity, or duration). These data suggest female mice have greater SD susceptibility, although this study did not examine the impact of estrus cycle stage [7]. In a subsequent study examining SD thresholds in familial hemiplegic migraine mutant mice expressing variants in the *CACNA1A* gene (S218L, R192Q), SD susceptibility (frequency with 300 mM topical KCl, and propagation speed) was higher in female mutants as compared to male mutants. Sex differences were not observed in WT mice [18]. Ovariectomy in the R192Q mutant abolished this sex difference which was partially restored following estradiol hormone replacement. These data implicate estradiol in increasing cortical excitability under genetically vulnerable conditions that would predispose females to SD [18]. Similarly, reduced susceptibility to SD in males may be attributed to gonadal hormones. In one study, orchiectomy increased SD susceptibility in male R192Q mutants which was reduced by testosterone pellet implantation [17]. In a recent study, ovariectomy abolished the increase in SD susceptibility observed in female rats, indicating circulating gonadal hormones indeed play a role in events upstream of trigeminal nociception [25].

In other trigeminal models, estrogens appear to increase nociception. In one study, repeated application of inflammatory soup (IS) to the dural surface via cannula produced greater decreases in periorbital mechanical threshold, locomotor activity, and time spent in the light in female ovariectomized mice treated with estradiol replacement as compared to those not treated with estradiol and as compared to males [42]. However, in this study, fluctuations in hormones in the physiological range encountered during normal cycling were not investigated. Similarly, estradiol replacement in ovariectomized rats produced greater light and noise avoidance behavior following dural IS administration and longer lasting facial grooming behavior, compared to corn oil vehicle control [46]. While estradiol produced worsened pain behavior in these studies, it is difficult to directly compare these results with periods during the estrus cycle. Nonetheless, one possible reason for why differences were seen in these prior studies and not with SD could be related to differences in the SD model versus IS migraine model. Alternatively, the unopposed action of estradiol may produce a pronociceptive phenotype, while in the normal cycling female, estradiol fluctuates along with other gonadal hormones including progesterone, luteinizing hormone, follicle stimulating hormone, gonadotropin releasing hormone and kisspeptin which may exacerbate or mitigate the effects of estradiol. Lastly, in these studies, estradiol was administered continuously, as opposed to the pulsed periodic endogenous production of estradiol that occurs across the normal estrus cycle.

Studies have used different methods to examine photosensitivity in animals including time spent in the lit area of a light–dark box. Alternatives include light induced grimace or squint [39]. While time spent in a light–dark box is a reliable measure of photosensitivity, it is also used to test anxiety [49] with recent studies that have attempted to address how to distinguish between the two [48]. Since our prior results demonstrate opto-SD induced anxiety-related behavior [24], and others have demonstrated freezing and reduced locomotor activity in light following opto-SD [35], we used grimace to reduce potential confounders in the interpretation of photophobia-like responses. There was increased grimace with increased light intensity for all groups except MD sham animals. Interestingly, following opto-SD, the grimace-light intensity curve was markedly upshifted. Our data suggest that, except for sham mice with presumably low circulating estrogens (i.e. those in MD), animals displayed some degree of photosensitivity. However, there was no effect of estrus cycle stage on SD-induced changes in the grimace-light intensity curve.

PE and MD groupings were used in this study to distinguish receptive female mice [13, 33] with high versus lower relative circulating estradiol [34, 41]. Grouping mice also increases statistical power and reduces the number of animals needed to study changes across the estrus cycle. To begin to determine if other approaches could be used to assist in determining cycle stage, IRT measurements of variations in surface temperature were investigated with vaginal lavage. Our data suggest that IRT surface temperature may be a useful tool to predict estrus cycle stage in animals but should be confirmed with vaginal lavage. This may be particularly useful when there is inadequate sampling across the cycle and may be used to increase sample size for stages of the cycle that are shorter duration without the need for repeated invasive handling and recurrent vaginal sampling methods that could induce pseudopregnancy. IRT has been used in larger animals and can be affected by other variables including room temperature, pain, and inflammation as well as body surface region tested [11]. Few studies have examined changes in surface temperature in mice with one study demonstrating lower temperatures during diestrus [38]. In sheep, one study recorded lower temperatures during diestrus as well using IRT [5]. IRT has also been proposed as a potential method for estrus and ovulation detection in cow and gilt [44, 45] with some differences in maximal temperature detection during different phases of the cycle. There are distinctions in the timing of changes in hormones between the human menstrual cycle and the mouse estrus cycle. In humans, the peak temperature occurs in the peri-ovulatory period, typically just after ovulation [9]. While it is difficult to extrapolate timing of peak temperatures in the human with temperature changes in mouse, ovulation occurs during the estrus phase of the mouse cycle [10]. Additional studies in larger cohorts may be needed to determine the sensitivity and specificity of this method as a noninvasive tool for prediction of estrus cycle stage in mice. In this study, we did not do standard interval assessment of estrus cycle stage or determine temperature at time of ovulation [38]. Nonetheless, like other reports, we did find that temperature changed during the estrus cycle in a small cohort of animals. This method may therefore be a promising non-invasive tool for future use.

There are several limitations in this study. First, while we used vaginal smear for estrus cycle stage, we did not collect serum samples from mice to do estradiol, progesterone or other serum hormone quantitative assays because of prior evidence detailing estradiol levels across the estrus cycle. However, it is possible that since we were randomly sampling animals and not doing daily vaginal smears that a small number of animals could be staged incorrectly. That we were able to see an effect of cycle stage in the sham animals may argue against this possibility. Second, we did not examine hindpaw allodynia to determine if the effect of cycle stage in sham animals was restricted to the trigeminal region and indeed it may be a more widespread response. However, the focus for these experiments was on migraine-relevant phenotypes. Third, additional consequences of cycle stage on timing of allodynia or recovery from SD, and on anxiety phenotypes were not tested. Lastly, ablation of gonadal hormones with gonadectomy and hormone replacement which is often used to test the effect of single hormones in isolation was not tested. Gonadal hormones tested in this way are often given in supraphysiologic doses and with continuous exposure rather than in the phasic pattern typically experienced during menses. Therefore, the effects may differ from the contribution of that hormone studied in isolation. Nonetheless, there is utility to examining hormones in isolation following gonadectomy as well as examining the influence of sex chromosomes.

## Conclusions

Taken together, our data support the conclusion that female gonadal hormones contribute to baseline pain thresholds and following opto-SD, female sex contributes to differences in periorbital allodynia. While differences were seen between estrus cycle stages in the sham control group, opto-SD stimulation could overcome these differences to produce similar magnitude trigeminal pain behavior irrespective of the estrus cycle stage. These data suggest that the greatest impact of estrus cycle may be acting upstream to influence SD susceptibility rather than downstream to influence pain behavior outcome after SD induction.

## Data Availability

Data presented herein generated during the current study are freely available from the corresponding author upon request.

## References

[1] Alarcon-Alarcon D, Cabanero D, de Andres-Lopez J, Nikolaeva-Koleva M, Giorgi S, Fernandez-Ballester G, Fernandez-Carvajal A, Ferrer-Montiel A (2022). TRPM8 contributes to sex dimorphism by promoting recovery of normal sensitivity in a mouse model of chronic migraine. Nat Commun.

[2] Araya EI, Turnes JM, Barroso AR, Chichorro JG (2020). Contribution of intraganglionic CGRP to migraine-like responses in male and female rats. Cephalalgia.

[3] Avona A, Burgos-Vega C, Burton MD, Akopian AN, Price TJ, Dussor G (2019). Dural calcitonin gene-related peptide produces female-specific responses in rodent migraine models. J Neurosci.

[4] Avona A, Mason BN, Burgos-Vega C, Hovhannisyan AH, Belugin SN, Mecklenburg J, Goffin V, Wajahat N, Price TJ, Akopian AN, Dussor G (2021). Meningeal CGRP-Prolactin Interaction Evokes Female-Specific Migraine Behavior. Ann Neurol.

[5] Barros de Freitas AC, Ortiz Vega WH, Quirino CR, Bartholazzi Junior A, Gomes David CM, Geraldo AT, Silva Rua MA, Cipagauta Rojas LF, Eustaquio de Almeida Filho J, Burla Dias AJ. Surface temperature of ewes during estrous cycle measured by infrared thermography. Theriogenology 2018;119:245–251. 10.1016/j.theriogenology.2018.07.015 30059884

[6] Bi RY, Meng Z, Zhang P, Wang XD, Ding Y, Gan YH. Estradiol upregulates voltage-gated sodium channel 1.7 in trigeminal ganglion contributing to hyperalgesia of inflamed TMJ. PLoS One 2017;12(6):e0178589. 10.1371/journal.pone.0178589 PMC5459440 28582470

[7] Brennan KC, Romero Reyes M, Lopez Valdes HE, Arnold AP, Charles AC (2007). Reduced threshold for cortical spreading depression in female mice. Ann Neurol.

[8] Burstein R, Yarnitsky D, Goor-Aryeh I, Ransil BJ, Bajwa ZH (2000). An association between migraine and cutaneous allodynia. Ann Neurol.

[9] Buxton CL, Atkinson WB (1948). Hormonal factors involved in the regulation of basal body temperature during the menstrual cycle and pregnancy. J Clin Endocrinol Metab.

[10] Byers SL, Wiles MV, Dunn SL, Taft RA (2012). Mouse estrous cycle identification tool and images. PLoS ONE.

[11] Alejandro Casas-Alvarado, Julio Martínez-Burnes, Patricia Mora-Medina, Ismael Hernández-Avalos, Adriana Domínguez-Oliva, Karina Lezama-García, et al (2022) Thermal and circulatory changes in diverse body regions in dogs and cats evaluated by infrared thermography. Animals (Basel) 12(6):789. 10.3390/ani12060789 10.3390/ani12060789 PMC8944468 35327185

[12] Chancellor AM, Wroe SJ, Cull RE (1990). Migraine occurring for the first time in pregnancy. Headache.

[13] Chari T, Griswold S, Andrews NA, Fagiolini M (2020). The stage of the estrus cycle is critical for interpretation of female mouse social interaction behavior. Front Behav Neurosci.

[14] Chung DY, Sugimoto K, Fischer P, Bohm M, Takizawa T, Sadeghian H, Morais A, Harriott A, Oka F, Qin T, Henninger N, Yaseen MA, Sakadzic S, Ayata C (2018). Real-time non-invasive in vivo visible light detection of cortical spreading depolarizations in mice. J Neurosci Methods.

[15] Cupini LM, Matteis M, Troisi E, Calabresi P, Bernardi G, Silvestrini M (1995). Sex-hormone-related events in migrainous females. A clinical comparative study between migraine with aura and migraine without aura. Cephalalgia.

[16] Deuis JR, Dvorakova LS, Vetter I (2017). Methods used to evaluate pain behaviors in rodents. Front Mol Neurosci.

[17] Eikermann-Haerter K, Baum MJ, Ferrari MD, van den Maagdenberg AM, Moskowitz MA, Ayata C (2009). Androgenic suppression of spreading depression in familial hemiplegic migraine type 1 mutant mice. Ann Neurol.

[18] Eikermann-Haerter K, Dilekoz E, Kudo C, Savitz SI, Waeber C, Baum MJ, Ferrari MD, van den Maagdenberg AM, Moskowitz MA, Ayata C (2009). Genetic and hormonal factors modulate spreading depression and transient hemiparesis in mouse models of familial hemiplegic migraine type 1. J Clin Invest.

[19] Elliott MB, Oshinsky ML, Amenta PS, Awe OO, Jallo JI (2012). Nociceptive neuropeptide increases and periorbital allodynia in a model of traumatic brain injury. Headache.

[20] Fata JE, Chaudhary V, Khokha R (2001). Cellular turnover in the mammary gland is correlated with systemic levels of progesterone and not 17beta-estradiol during the estrous cycle. Biol Reprod.

[21] Yesenia Garcia-Sifuentes, Donna L Maney (2021) Reporting and misreporting of sex differences in the biological sciences. Elife 10:e70817. 10.7554/eLife.70817 10.7554/eLife.70817 PMC8562995 34726154

[22] Granella F, Sances G, Pucci E, Nappi RE, Ghiotto N, Napp G (2000). Migraine with aura and reproductive life events: a case control study. Cephalalgia.

[23] Granella F, Sances G, Zanferrari C, Costa A, Martignoni E, Manzoni GC (1993). Migraine without aura and reproductive life events: a clinical epidemiological study in 1300 women. Headache.

[24] Harriott AM, Chung DY, Uner A, Bozdayi RO, Morais A, Takizawa T, Qin T, Ayata C (2021). Optogenetic spreading depression elicits trigeminal pain and anxiety behavior. Ann Neurol.

[25] Kudo C, Harriott AM, Moskowitz MA, Waeber C, Ayata C (2023). Estrogen modulation of cortical spreading depression. J Headache Pain.

[26] Lee H, Iida T, Mizuno A, Suzuki M, Caterina MJ (2005). Altered thermal selection behavior in mice lacking transient receptor potential vanilloid 4. J Neurosci.

[27] Lipton RB, Bigal ME, Ashina S, Burstein R, Silberstein S, Reed ML, Serrano D, Stewart WF (2008). American Migraine Prevalence Prevention Advisory G. Cutaneous allodynia in the migraine population. Ann Neurol.

[28] Lipton RB, Bigal ME, Diamond M, Freitag F, Reed ML, Stewart WF, Group AA (2007). Migraine prevalence, disease burden, and the need for preventive therapy. Neurology.

[29] Lipton RB, Fanning KM, Buse DC, Martin VT, Reed ML, Manack Adams A, Goadsby PJ (2018). Identifying natural subgroups of migraine based on comorbidity and concomitant condition profiles: Results of the Chronic Migraine Epidemiology and Outcomes (CaMEO) Study. Headache.

[30] LoPinto C, Young WB, Ashkenazi A (2006). Comparison of dynamic (brush) and static (pressure) mechanical allodynia in migraine. Cephalalgia.

[31] Lovati C, D'Amico D, Rosa S, Suardelli M, Mailland E, Bertora P, Pomati S, Mariani C, Bussone G (2007). Allodynia in different forms of migraine. Neurol Sci.

[32] MacGregor EA (2004). Oestrogen and attacks of migraine with and without aura. Lancet Neurol.

[33] Martin VT, Lee J, Behbehani MM (2007). Sensitization of the trigeminal sensory system during different stages of the rat estrous cycle: implications for menstrual migraine. Headache.

[34] McLean AC, Valenzuela N, Fai S, Bennett SA (2012). Performing vaginal lavage, crystal violet staining, and vaginal cytological evaluation for mouse estrous cycle staging identification. J Vis Exp.

[35] Pi C, Tang W, Li Z, Liu Y, Jing Q, Dai W, Wang T, Yang C, Yu S (2022). Cortical pain induced by optogenetic cortical spreading depression: from whole brain activity mapping. Mol Brain.

[36] Pradhan AA, Smith ML, McGuire B, Tarash I, Evans CJ, Charles A (2014). Characterization of a novel model of chronic migraine. Pain.

[37] Puri V, Cui L, Liverman CS, Roby KF, Klein RM, Welch KM, Berman NE (2005). Ovarian steroids regulate neuropeptides in the trigeminal ganglion. Neuropeptides.

[38] Ratko M, Habek N, Kordic M, Dugandzic A (2020). The use of infrared technology as a novel approach for studies with female laboratory animals. Croat Med J.

[39] Rea BJ, Wattiez AS, Waite JS, Castonguay WC, Schmidt CM, Fairbanks AM, Robertson BR, Brown CJ, Mason BN, Moldovan-Loomis MC, Garcia-Martinez LF, Poolman P, Ledolter J, Kardon RH, Sowers LP, Russo AF (2018). Peripherally administered calcitonin gene-related peptide induces spontaneous pain in mice: implications for migraine. Pain.

[40] Rossi HL, Lara O, Recober A (2016). Female sex and obesity increase photophobic behavior in mice. Neuroscience.

[41] Saleeon W, Jansri U, Srikiatkhachorn A, Bongsebandhu-Phubhakdi S (2015). The estrous cycle modulates voltage-gated ion channels in trigeminal ganglion neurons. J Physiol Sci.

[42] Eleonóra Spekker, Zsuzsanna Bohár, Annamária Fejes-Szabó, Mónika Szűcs, László Vécsei, Árpád Párdutz (2022) Estradiol treatment enhances behavioral and molecular changes induced by repetitive trigeminal activation in a rat model of migraine. Biomedicines 10(12):3175. 10.3390/biomedicines10123175 10.3390/biomedicines10123175 PMC9776064 36551931

[43] Stucky NL, Gregory E, Winter MK, He YY, Hamilton ES, McCarson KE, Berman NE (2011). Sex differences in behavior and expression of CGRP-related genes in a rodent model of chronic migraine. Headache.

[44] Sykes DJ, Couvillion JS, Cromiak A, Bowers S, Schenck E, Crenshaw M, Ryan PL (2012). The use of digital infrared thermal imaging to detect estrus in gilts. Theriogenology.

[45] Talukder S, Kerrisk KL, Ingenhoff L, Thomson PC, Garcia SC, Celi P (2014). Infrared technology for estrus detection and as a predictor of time of ovulation in dairy cows in a pasture-based system. Theriogenology.

[46] Vermeer LM, Gregory E, Winter MK, McCarson KE, Berman NE (2015). Behavioral effects and mechanisms of migraine pathogenesis following estradiol exposure in a multibehavioral model of migraine in rat. Exp Neurol.

[47] Viero FT, Rodrigues P, Frare JM, Da Silva NAR, Ferreira MA, Da Silva AM, Pereira GC, Ferreira J, Pillat MM, Bocchi GV, Nassini R, Geppetti P, Trevisan G (2022). Unpredictable sound stress model causes migraine-like behaviors in mice with sexual dimorphism. Front Pharmacol.

[48] Mengya Wang, Bianca N Mason, Levi P Sowers, Adisa Kuburas, Brandon J Rea, Andrew F Russo (2021) Investigating migraine-like behavior using light aversion in mice. J Vis Exp (174):10.3791/62839. 10.3791/62839 10.3791/62839 PMC8428768 34459825

[49] Young R, Johnson DN (1991). A fully automated light/dark apparatus useful for comparing anxiolytic agents. Pharmacol Biochem Behav.

